# A Carbon Composite Film with Three-Dimensional Reticular Structure for Electromagnetic Interference Shielding and Electro-Photo-Thermal Conversion

**DOI:** 10.3390/ma14092423

**Published:** 2021-05-06

**Authors:** Na Lin, Hanning Chen, Xiaokang Mei, Shitong Chai, Longsheng Lu

**Affiliations:** 1 School of Mechanical Engineering, Tianjin Polytechnic University, Tianjin 300387, China; linna8105@hotmail.com (N.L.); chenhanning@tjpu.edu.cn (H.C.); 2 School of Computer Science and Software, Tianjin Polytechnic University, Tianjin 300387, China; 3 School of Mechanical & Automotive Engineering, South China University of Technology, Guangzhou 510641, China; cst0355@163.com (S.C.); meluls@scut.edu.cn (L.L.)

**Keywords:** electromagnetic interference shielding, carbon fabric, graphene, electro-thermal conversion, photo-thermal effect

## Abstract

The design of flexible wearable electronic devices that can shield electromagnetic waves and work in all weather conditions remains a challenge. We present in this work a low-cost technology to prepare an ultra-thin carbon fabric–graphene (CFG) composite film with outstanding electromagnetic interference shielding effectiveness (EMI SE) and electro-photo-thermal effect. The compatibility between flexible carbon fabric skeleton and brittle pure graphene matrix empowers this CFG film with adequate flexibility. The reticular fibers and porous structures play a vital role in multiple scattering and absorption of electromagnetic waves. In the frequency range of 30–1500 MHz, the CFG film can achieve a significantly high EMI SE of about 46 dB at tiny thickness (0.182 mm) and density (1.4 g cm^−3^) predominantly by absorption. At low safe voltages or only in sunlight, the film can self-heat to its saturation value rapidly in 40 s. Once the electricity or light supply is stopped, it can quickly dissipate heat in tens of seconds. A combination of the EMI SE and the prominent electro-photo-thermal effect further enables such a remarkable EMI shielding film to have more potential applications for communication devices in extreme zones.

## 1. Introduction

Mobile communication creates a revolution in the telecommunication industry, extending network terminals from fixed locations to individuals. People can process and transmit information whenever and wherever possible through mobile devices. Contemporarily, the world is accelerating the pace of 5G commercial use. For the higher demand for mobile data, seamless and deep coverage of 5G networks relies more on the deployment of miniaturized base stations [1]. During their construction and operation, electromagnetic radiation that is concerned with public health has become the focus of people’s attention. In addition, electromagnetic interference (EMI) between electronic components inside the base station mightily impedes their functional operation [2].

To this end, EMI shielding materials play an important role in electromagnetic protection of the base stations. Usually, base station housing is made of aluminum alloy and other metal die casting with high EMI shielding effectiveness (EMI SE). These metal materials, however, suffer from the drawbacks of having high density, poor flexibility, and weak corrosion resistance. Owing to light mass density, sufficient mechanical flexibility, adequate corrosion resistance, and favorable shaping capability, conductive polymer composites (CPCs) are good alternative materials widely employed in the field of EMI shielding [3]. Carbon materials not only have excellent physical and chemical properties, but also do not cause too much harm to the environment, such as grapheme [4,5,6,7], carbon black [8,9], multi-walled carbon nanotubes [10,11] and so on. Porous carbon materials are the best choice to resist electromagnetic shielding interference due to their light weight, large specific surface area, corrosion resistance, high electrical and thermal conductivity. Therefore, adding carbon materials to CPCs to improve their conductivity and enhance the electromagnetic shielding performance has become a research hotspot in recent years. Octavio Alejandro Castañeda-Uribe et al. controlled the distribution of the carbon nanotubes by applying a sinusoidal AC voltage to enhance electromagnetic shielding performance (EMI SE 77 dB) for single-walled carbon nanotubes/polyimide (SWCNT/PI) nanocomposite films [12]. Ziming Shen et al. backfilled epoxy into wood-derived carbon scaffolds to acquire high electromagnetic interference shielding effectiveness (EMI SE 27.8 dB) and thermal conductivity (TC) [13]. Weiwei Gao et al. produced graphene/polymer composites with a highly arranged network structure that exhibited anisotropic conductivity and mechanical properties at very low graphene content, and thus had significant electromagnetic shielding performance (EMI SE 65 dB) [14].

Although significant efforts of CPCs have been devoted to electromagnetic protection of base stations, the only fly in the ointment is that the outdoor working conditions are often fickle. Sometimes there are awfully severe environments, such as broiling or freezing environments, and the performance of CPCs will deteriorate after prolonged exposure. Researchers tend to pay more attention to the multifunctional integration of EMI shielding CPCs, while rarely does the literature report on solutions to the failure of CPCs in extreme environments. As everyone knows, there is a large temperature difference between day and night in plateau or desert areas. When the ambient temperature is very high during the day, the heat dissipation capacity of polymers is far from sufficient. When the ambient temperature at night is lower than the glass transition temperature of polymers, they will become unusually brittle [15]. All of these restrict the practical application of CPCs in harsh conditions. Therefore, it is expected that CPCs take on a quality of rapid heating and cooling, guaranteeing their flexibility or normal functions at extreme temperatures.

Herein, we demonstrate a flexible carbon fabric–graphene (CFG) composite film with exceptional EMI SE and electro-photo-thermal effect. Based on our previous work [6], the porous structure formed by the fusion of graphene and polyvinylidene fluoride (PVDF) is utilized to construct an electromagnetic energy dissipation network and a heat conduction path. The introduction of flexible carbon fabric, consisting of carbon fibers (CFs) and polypropylene/polyethylene (PP/PE) core/sheath bicomponent fibers (ESFs), greatly reduces the brittleness of graphene and PVDF matrix. Furthermore, compared with other electromagnetic shielding materials, it is found that the CFG film manages prominent electro-photo-thermal effects. Therefore, it is possible to apply external energy to the CFG film to enable it to work in extremely cold environment.

## 2. Materials and Methods

The materials and parameters used are listed in Table 1.

**Figure 1:**
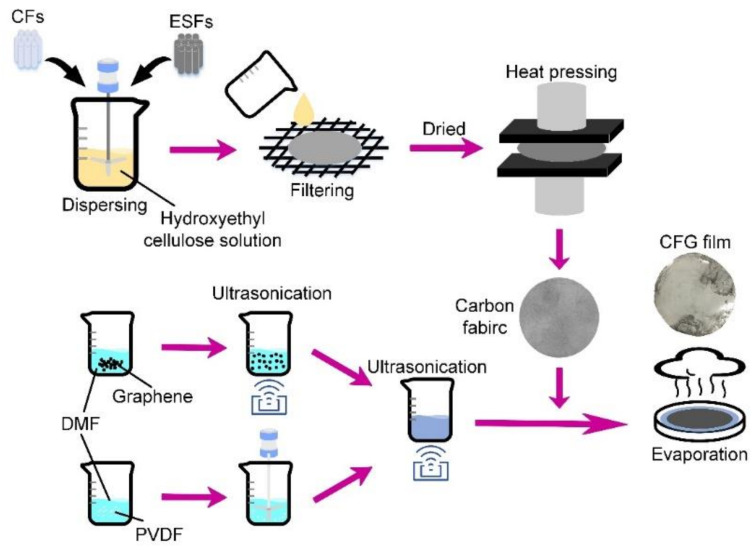
Schematic diagram of the preparation process of a CFG film.

As shown in Figure 1, the whole preparation process of a CFG film mainly includes the following three steps:

(i) To prepare a carbon fabric, first, the pre-prepared ESFs and chopped CFs, both with a length of 6 mm, were mixed and dispersed in hydroxyethyl cellulose solution. Then, the mixed solution was stirred for 4 min at 700 rpm and stood for 5 min. After that, the mixed stable solution was filtered via stainless steel mesh (#80) to obtain a wet preformed carbon fabric, which was next dried at 80 °C for 30 min. Afterwards, the preformed carbon fabric was heat-pressed to form a flexible and robust carbon fabric [16] using a curing press at 170 °C and 6 MPa.
(ii) To prepare a homogenized solution of graphene and PVDF, first of all, graphene nanoplatelets and PVDF powders, respectively, were weighed and added in DMF solvent. The PVDF/DMF solution was treated by ultrasonication at 60 °C for 6 h to get evenly dispersed, and the graphene/DMF solution was mechanically stirred at 60 °C for 2 h. Then, they are mixed and treated in the same way as the dispersion of graphene/DMF solution.
(iii) To prepare a CFG film, in the beginning, the pre-dried carbon fabric was immersed into the homogenized solution of graphene and PVDF. Later, the resulting mixture was transferred to a Petri dish with a non-stick polyimide film on the bottom. Subsequently, the Petri dish containing the mixture was put in an oven at 80 °C for about 18 h to remove all DMF. During evaporation, the mixture should be transferred to a new container in time to avoid the difference in moisture between upper and lower layers, which may cause the upper and lower layers to contract and expand at different degrees, resulting in the final film wrinkling. Finally, an ultra-thin CFG film can be taken off from the polyimide film [17].

A field emission scanning electron microscope (SEM, ZEISS Merlin, Jena, Germany) was used to observe the surface and cross-section morphology of the CFG film that had been cryogenically fractured via liquid nitrogen. Transmission electron microscopy (TEM, FEI Tecnai G2 F20, FEI NanoPorts, Hillsboo, OR, USA) was used to verify the dispersion of graphene in the PVDF matrix. The EMI shielding measurement was carried out with the help of a network analyzer (NA7500, Tianjin Deli Electricity Company, Tianjin, China) with shielding effectiveness test equipment (model DR-S01, Beijing DR Technology Co., Ltd, Beijing, China). A DC power supply (MS605D, MAISHENG, Dongguan, China) was used as the electric energy source for the electro-thermal effect of the CFG film. An illuminometer was used to measure the illuminance of light. The temperature of the CFG film was recorded in real time by an infrared thermal imager (ThermaCAM SC3000, Celiss, Tianjin, China). An electronic balance (XSE104, METTLER TOLEDO, Zurich, Switzerland) served for weighing. The thickness of the CFG film was measured using a paper thickness tester (IMT-HD01, INTENAI, Dongguan, China). All dimensions were measured directly by steel ruler (BONTHE, Taizhou, China) whose precision was ±0.1 cm.

## 3. Results and Discussion

### 3.1. Morphological Observation

**Figure 2:**
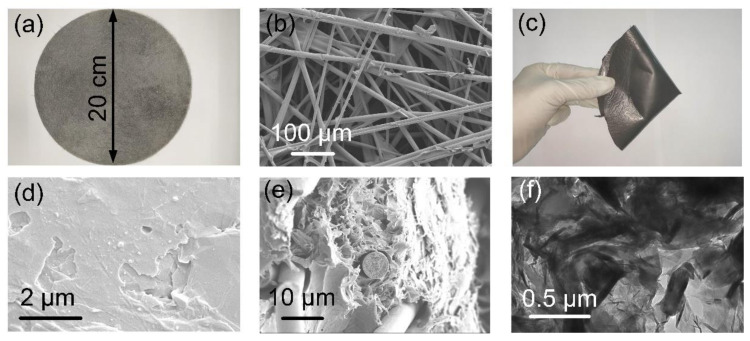
(**a**) Macroscopic and (**b**) microscopic morphology of a flexible and robust carbon fabric. (**c**) Folding the CFG film shows its flexibility. (**d**) Surface morphology and (**e**) cross-section morphology of the CFG film. (**f**) Transmission electron microscopy of ultrathin slices of the CFG film.

Figure 2a,b show the macroscopic and microscopic morphology of a flexible carbon fabric. The strength of the entire carbon fabric is provided by hot-pressing nodes formed by melting and cooling of the PE shell of the ESFs. Strong nodes help the CFs to maintain a stable and uniform regional distribution in the subsequent homogenized solution of graphene and PVDF. In order to withstand complex deformations, such as folding, bending and torsion in practical applications, EMI films are expected to possess satisfactory mechanical flexibility. The introduction of a flexible carbon fabric helps to lap a flexible skeleton in a brittle graphene/PVDF matrix, rendering the CFG film sufficient flexibility, as displayed in Figure 2c–e, which shows, respectively, the surface morphology and internal structure of the CFG film. It is found that the surface of the CFG film is relatively flat, but the inside of the graphene/PVDF matrix presents a porous-layered network where fibers are randomly embedded. Figure 2f verifies the fair distribution and connection of graphene in PVDF, creating a favorable conductive network (2.7 S cm^−1^) that is regarded as the most critical parameter in governing the EMI shielding characteristics [18].

### 3.2. EMI Shielding Performance

According to ASTM D4935-10 standard, the scattering parameters (S_11_ and S_21_) of the CFG film in a 30–1500 MHz frequency range were evaluated. From the measured reflection scattering parameters (S_11_) and absorption scattering parameters (S_21_), the reflection coefficient (R) and transmission coefficient (T) and absorption coefficient (A) can be calculated according to the following formula:

(1) $$R={\left|S_{11}\right|}^{2}$$

(2) $$T={\left|S_{21}\right|}^{2}$$

(3) A=1 − R − T

The EMI SE (SE_T_) consists of three parts: reflection loss (SE_R_), absorption loss (SE_A_) and multiple reflection loss (SEM), expressed by the following formula:

(4) $${\mathrm{EMI}\;\mathrm{SE}=\mathrm{SE}}_{R}{+\mathrm{SE}}_{A}{+\mathrm{SE}}_{M}$$

where SE_M_ and SE_A_ are associated. When SE_T_ > 15 dB, SEM tends to be ignored. In that way, with the calculated R, T and A, SE_T_, SE_R_ and SE_A_ can be determined as follows:

(5) $${\mathrm{SE}}_{T}=10\log\left|\frac{1}{T}\right|{=\mathrm{SE}}_{A}{+\mathrm{SE}}_{R}$$

(6) $${\mathrm{SE}}_{R}=10\log\frac{1}{1\;-\;R}=10\log\frac{1}{1\;-\;{\left|S_{11}\right|}^{2}}$$

(7) $${\mathrm{SE}}_{A}=10\log\frac{1\;-\;R}{T}=10\log\frac{1\;-\;{\left|S_{11}\right|}^{2}}{{\left|S_{21}\right|}^{2}}$$

**Figure 3:**
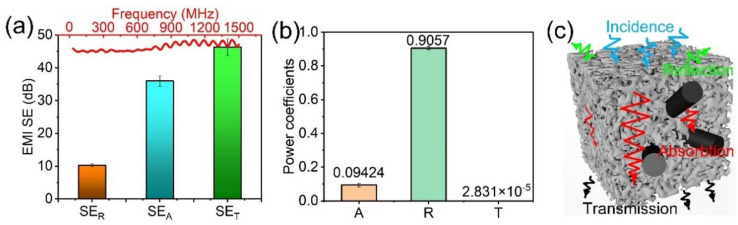
(**a**) EMI SE, (**b**) power coefficients, and (**c**) EMI shielding mechanism of the CFG film.

Figure 3a suggests that the CFG film with a thickness of 0.182 mm, diameter of 20 mm and a density of 1.4 g cm^−3^ possesses an excellent EMI SE of about 46 dB, divided into the SE_A_ of 35.71 dB and the SE_R_ of 10.29 dB. Our data mean that the CFG film can shield over 99.997% of incoming wave energy, mainly ascribing attenuation by absorption. Meanwhile, the commercial standard required for typical EMI materials is 20 dB, corresponding to 99% attenuation of incident energy [19]. Figure 3b illustrates the absorption coefficient (A), reflection coefficient (R) and transmission coefficient (T) of the CFG film, which represent its actual shielding ability. It can be found that the value of R and A are 0.9057 and 0.09424, respectively, while the value of T is close to 0. This is due to the fact that graphene, which is highly conductive on the surface of the CFG film, first reflects a lot of electromagnetic waves. The electromagnetic waves that enter the CFG film are almost exhausted.

Essential EMI shielding mechanism of the CFG film is indicated in Figure 3c. The incident waves are initially reflected by the CFG film due to the discontinuity of impedance at their interface. Electromagnetic waves that enter CFG film are attenuated by either internal conduction path or multiple scattering in porous structure [20], both promoting the absorption loss of electromagnetic energy. This is consistent with the observation that SE_A_ predominates over SE_R_. Eventually, little waves pass through the CFG film.

### 3.3. Electro-Photo-Thermal Effect

**Figure 4:**
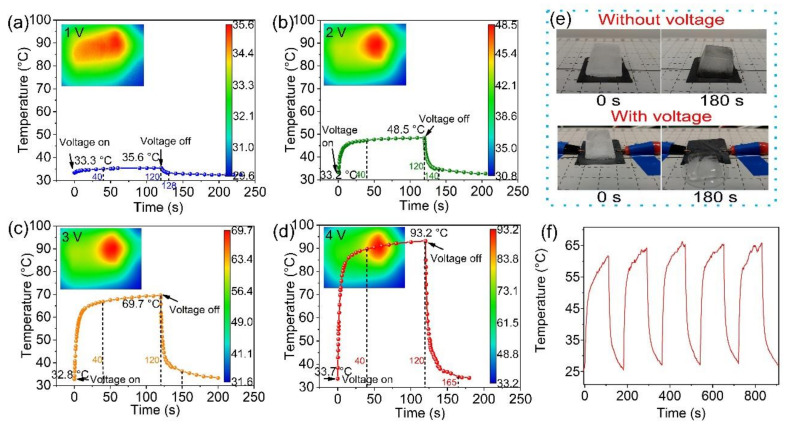
Electro-thermal response of the CFG film (30 × 30 mm^2^) applied with voltages of (**a**) 1 V, (**b**) 2 V, (**c**) 3 V, and (**d**) 4 V. (**e**) Comparison of rapid deicing effect of the CFG film without and with voltage (4 V). (**f**) The repeated experiment at 3 V.

As can be seen from Figure 4a–d, the CFG film exhibits a rapid electro-thermal response. During the experiment, the temperature of electrode chuck at one end of the film had an effect on infrared temperature measurement. In addition, the thin thickness of the film led to deformation when the two ends were clamped. So, we shifted the position of the CFG film, then the thermal center of the CFG film is not in the center of the figure. Moreover, the temperature of the CFG film increases rapidly with the voltage and time, and then approaches an equilibrium value after 40 s. Applying voltages of 1, 2, 3, and 4 V severally for 2 min, the center temperature of the CFG film can reach 35.6, 48.5, 69.7, and 93.2 °C, respectively. When cutting off the voltage, it only separately takes 8, 20, 30, and 45 s to cool to local room temperature. The conductivity of the CFG film measured by a four-probe tester is 2.7 S cm^−1^. Therefore, the Joule-heating efficiency can be expressed by the following formula:

(8) $$P=\frac{V^{2}}{Rs}$$

where *P* is the power, *V* is the voltage, and *Rs* is the sheet resistance. The power density (*W*) can be calculated by the following equation:

(9) $$W=\frac{P}{S}$$

where *P* is heating power, and *S* is the area of the surface of CFG film. The power density in this paper could reach 873.6 W/m^2^.

As is well known, the mechanical performance and EMI shielding property of the ice-covered CPCs will be greatly compromised. Due to the remarkable electro-thermal response, the CFG film is easily qualified with high-speed deicing capability [3]. In Figure 4e, at room temperature (24 °C), an ice cube in the shape of frustum of a pyramid about 6.120 g (upper bottom size 16 × 16 mm^2^, lower bottom size 21 × 21 mm^2^, height 19 mm) on the CFG film melted after 3 min with the remaining 5.172 g (15.49% melted at room temperature). When a voltage of 4 V was supplied, the bottom of the ice cube (6.067 g) began to melt, leaving a residue of 2.427 g after 3 min (60.00% melted). Due to the low water adhesion of the CFG film brought by PVDF, the melting ice will automatically slide off. The principle of the CFG film’s electro-thermal response can be explained as follows: Under the action of electric field, free electrons within the CFG film will be oriented through conductive network. During the directional flow of electrons, they constantly collide with other electrons, accelerating the motion of these electrons and increasing the internal energy. In consequence, the conversion of electrical energy to internal energy is realized. In Figure 4f, we carried out the repeated experiment at 3 V. It can be found that the temperature curve is consistent, which proves the stability of the CFG film.

**Figure 5:**
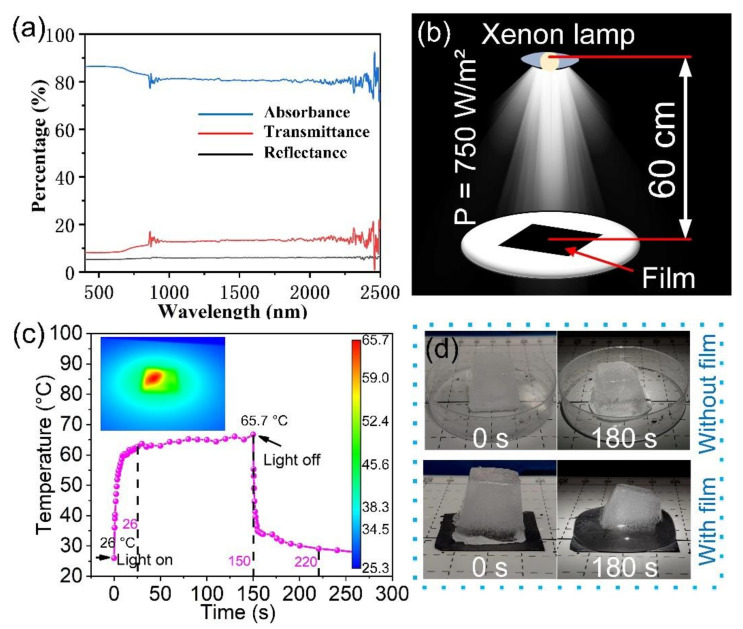
(**a**) Vis-NIR absorbance, reflectance, and transmittance of CFG film. (**b**) Diagram of photo-thermal apparatus for testing photo-thermal response of the CFG film. (**c**) Photo-thermal response of the CFG film (30 × 30 mm^2^). (**d**) Comparison of fast deicing effect of the CFG film without and with film.

Intriguingly, except for the electro-thermal response, a desired photo-thermal response is achieved under light exposure for a short time. As shown in Figure 5a, the Vis-NIR absorption spectrum is the main factor to evaluate the capacity of optical absorption for deicing. The light absorption performance of the CFG film was studied by visible and near IR-range absorbance, reflectance, and transmittance measurement in the wavelength range of 400–2500 nm. Except for the interference at 800 and 2500 nm, it can be found that the average absorbance of CFG film reaches 81.55% from 1000 to 2000 nm. In addition, the absorbance of CFG film also reached more than 80% in visible light range. The results show that CFG film has low reflectance and transmittance, and excellent wide band light absorption. The reason is that the CFG film has strong and wide band light absorbance and its porous structures possess the multiple scattering effect. Then, we set up the photo-thermal experiment device as depicted in Figure 5b, in which the xenon lamp was used to simulate sunlight. When the CFG film is irradiated, the photonic energy interacts with the crystal lattice, intensifying the vibration and increasing the temperature [21]. Figure 5c reveals that under the light irradiation of 750 W m^−2^, the CFG film approached the temperature saturation point (65.7 °C) in only 26 s and dropped to 35 °C in 5 s once the light was turned off. Figure 5d estimates that the photo-thermal effect of the CFG film on deicing under the same light radiation condition. When placed in a plastic petri dish, 6.096 g of the ice cube melted to 4.981 g (18.29% melted). However, the CFG film can melt 6.146 g of an ice cube by about half in 3 min, leaving 3.107 g.

In order to meet the needs of portable electronic devices operating at high performance and low power levels in the 5G era, the ability of the CFG film to quickly heat up with low power consumption is also required. Based on this, we propose specific electro-thermal efficiency and specific photo-thermal efficiency. Specific electro-thermal efficiency (indicated with *Sete*, °C V^−1^ s^−1^) is defined as: the temperature difference ΔT (°C) when the temperature rises to 95% of the equilibrium value within the time t (s) under the voltage U (V). Table 2 lists and compares the *Sete* of some recent electro-thermal materials and the CFG film. Although the heat change of CFG film is not the largest in Table 2, it can rise to a higher temperature at a lower voltage and in a shorter time. Specific photo-thermal efficiency (indicated with *Spte*, °C W^−1^ m^2^ s^−1^) is defined as: the temperature difference ΔT (°C) when the temperature rises to 95% of the equilibrium value within the time t (s) under the irradiance E (W m^−2^). The *Spte* of some typical photo-thermal materials and the CFG film are listed and compared in Table 3. As shown in Table 3, CFG films can raise to higher temperatures in a shorter period of time with lower light energy compared with others. The larger the values of *Sete* and *Spte*, the more prominent the electro-photo-thermal effect of the material. It can be discovered that the *Sete* and *Spte* of the CFG film are significantly higher than that of the recent competitive materials, which indicates that the CFG film can achieve better electro-photo-thermal conversion at a lower power level.

Therefore, the integration of outstanding low-voltage-driven electro-thermal response and easily accessible photo-thermal response, as well as high EMI shielding performance, may further extend the potential applications of the CFG film. The strategy of the CFG film is expected to be a low-hanging technical solution to the performance degradation of communication equipment in extreme temperature environments.

## 4. Conclusions

A flexible 0.182 mm thick CFG film manifests exceptional EMI SE (up to 46 dB) over a range of 30–1500 MHz. The internal porous conductive network promotes the absorption loss of electromagnetic energy. At a low safe voltage (4 V) or under readily available sunlight (750 W/m^2^), this film is capable of rapid self-heating and self-cooling in tens of seconds. Superadding water resistance of PVDF matrix, snow or ice on film can be removed effortlessly. This work may open a new avenue for multifunctional next-generation wearable electronics used in polar regions.

## Figures and Tables

**Table 1:** Materials used and related parameters in this work.

	Materials	Mass Fraction		Notes
CFG film	Carbon fabric	8.6 wt%, 40 gsm	CFs 40 wt%	Polyacrylonitrile (PAN)-based CFs
			ESFs 60 wt%	Composed of PE sheath (melting region: 130–150 °C) and PP core (melting region: 180–200 °C)
	PVDF	51.4 wt%		Forming a porous lamellar matrix
	Graphene	40 wt%		
Auxiliary materials	Hydroxyethyl cellulose solution	1.2 wt%		A dispersion agent for CFs and ESFs
	N, N-dimethylformamide (DMF)	Moderate		A solvent for PVDF powder and graphene

**Table 2:** Comparison of the *Sete* of some recent electro-thermal materials and the CFG film.

Materials	ΔT (°C)	U (V)	t (s)	*Sete* (°C V^−1^ s^−1^)	Ref.
Graphene ink on A4-size paper or polyester	50.2	10	30	0.1673	[22]
Graphene/tourmaline composite fabric	63.8	10	40	0.1595	[23]
C22@GO–CNT microcapsules	57.5	6	300	0.03194	[24]
ANFs/CNTs hybrid aerogel films	113.5	10	40	0.2837	[25]
MXene-decorated cotton fabrics	65	4	50	0.3250	[26]
CFG film	67.2	4	40	0.4200	This work

**Table 3:** Comparison of the *Spte* of some typical photo-thermal materials and the CFG film.

Materials	ΔT (°C)	E (W m^−2^)	t (s)	*Spte* (°C W^−1^ m^2^ s^−1^)	Ref.
Carbon foam-based phase change composites	26.3	870	100	3.02 × 10^−4^	[27]
FeS_2_ nanodots	29	800	300	1.21 × 10^−4^	[28]
Carbon black	18.5	1000	1800	1.02 × 10^−5^	[29]
WO_3−x_/C nanosheet	154	2000	200	3.85 × 10^−4^	[30]
Pyroelectric polymer films	16.7	1640	20	5.09 × 10^−4^	[31]
CFG film	39.7	750	26	2.04 × 10^−3^	This work

## Data Availability

The data presented in this study are available on request from the corresponding author. The data are not publicly available due to privacy restrictions.
